# High-resolution automated mapping of potential *Aedes* larval container habitats using drone imagery and supervised machine learning in Dar es Salaam, Tanzania

**DOI:** 10.1371/journal.pntd.0014361

**Published:** 2026-05-26

**Authors:** Mary Hahm, Venkanna Babu Guthula, Remigio Chilaule, Dimitri Gominski, Alex Limwagu, Exavery Chaki, Fredros Okumu, Leka Tingitana, Anders Hermund, Lucy S. Tusting, Rasmus Fensholt, Gustavo Riberio, Jakob Brandtberg Knudsen, Johan Mottelson, Yeromin Mlacha, Mary Cameron, Christian Igel

**Affiliations:** 1 Department of Control of Diseases, London School of Hygiene and Tropical Medicine, London, United Kingdom; 2 Department of Computer Science, University of Copenhagen, Copenhagen, Denmark; 3 Royal Danish Academy - Architecture, Design, Conservation, Copenhagen, Denmark; 4 Faculdade de Arquitectura e Planeamento Físico, Universidade Eduardo Mondlane, Maputo, Mozambique; 5 Department of Geosciences and Natural Resource Management, University of Copenhagen, Copenhagen, Denmark; 6 Environmental Health and Ecological Science Department, Ifakara Health Institute, Mikocheni, Dar es Salaam, Tanzania; 7 School of Biodiversity, One Health and Veterinary Medicine, University of Glasgow, Glasgow, United Kingdom; 8 Tanzania Flying Labs, Dar es Salaam, Tanzania‌‌; Radboud University Nijmegen Radboud Institute for Molecular Life Sciences: Radboud Universiteit Radboud Institute for Molecular Life Sciences, NETHERLANDS, KINGDOM OF THE

## Abstract

Larval source management is a key strategy to control the spread of *Aedes*-borne viral diseases including dengue, Zika, chikungunya, and yellow fever. However, locating potential larval habitats through traditional field methods is challenging and labor-intensive at scale. Here, we demonstrate a scalable, high-resolution drone imagery and supervised machine learning approach to map potential *Aedes* larval container habitats across Dar es Salaam, Tanzania, a dense urban environment with informal settlements. Local larval surveillance and existing literature confirm epidemiological relevance of buckets and jerry cans, tires, and water tanks as key potential habitats. Drone images revealed rooftop tires, a container type likely overlooked during ground surveillance. We trained a U-Net deep learning model on very-high-resolution drone imagery (3–5 cm resolution) which was manually annotated across 4.6 km^2^, and applied it to predict containers across 27.27 km^2^, spanning 20 neighborhoods. The model predicted over 135,000 containers with detection accuracies of 75% for water tanks, 72% for tires, and 54% for buckets. Bucket and tire densities were strongly and positively correlated with population density across neighborhoods, whereas water tank density was not, suggesting the distribution of these container types reflect distinct underlying drivers. This study reveals otherwise difficult-to-observe container types, highlights the abundance and spatial heterogeneity of potential *Aedes* larval habitats across Dar es Salaam, and demonstrates a scalable approach for improving detection of potential *Aedes* larval container habitats in a dense urban environment.

## Author summary

Mosquito-borne diseases like dengue and Zika are increasingly a public health threat in African cities, driven by *Aedes* mosquitoes that breed in small water-holding containers scattered across densely populated neighborhoods. Controlling these mosquitoes requires finding and eliminating their larval habitats, but doing this on the ground in large, complex cities is slow, labor-intensive and often incomplete. We started by analyzing field surveillance data from Dar es Salaam, Tanzania, and found that *Aedes* larvae were more likely to be found in buckets, tires, and water storage tanks than other types of habitats with larvae present. We flew drones over neighborhoods across the city to capture detailed aerial images and labeled all of the visible, relevant containers in a subsection of the imagery. We then developed a machine learning model to automatically identify these containers from unlabeled drone imagery. The model performed with up to 75% accuracy. This study demonstrates the feasibility of identifying and mapping visible container types using high-resolution drone imagery and machine learning in urban settings like Dar es Salaam.

## Introduction

Mosquito-borne viral diseases including dengue, chikungunya, yellow fever, and Zika pose significant public health threats in tropical and subtropical regions. These viruses, transmitted by *Aedes aegypti* and *Aedes albopictus* mosquitoes, cause major outbreaks and are increasingly a concern due to rapid urbanization, climate change, changes in vector ecology, and rising insecticide resistance [1–5]. In many endemic areas, diagnostic capacity and treatment options are limited, making vector control a key strategy for reducing disease burden through prevention [2,6].

In sub-Saharan Africa, where *Aedes* mosquitoes typically demonstrate exophilic behavior (outdoor biting and resting), standard vector control methods that prevent exposure to adult mosquitoes indoors, like bed nets and indoor residual spraying, are less applicable [7,8]. Larval source management (LSM), which encompasses larvicide application and source reduction by covering, cleaning, emptying or removing potential larval habitats, is therefore particularly important in these settings [7–10]. Effective larval source reduction relies on the ability to locate larval habitats, a challenging prerequisite in densely populated urban areas with informal settlements, pollution, suboptimal waste water drainage, and limited infrastructure [11–13].

In Dar es Salaam, Tanzania, *Aedes* mosquitoes tend to breed in small, man-made habitats including car tires, flowerpots, and small plastic containers [14–16]. Where traditional ground surveys would be labor-intensive and impractical, remote sensing technology presents an opportunity to detect objects at scale [17]. While open-source satellite imagery lacks the resolution to detect containers 10–50 cm in diameter, drones can capture images at resolutions up to 1 cm [18,19]. Drone imagery has been established as a tool for identifying potential *Anopheles* larval habitats, primarily natural water bodies like ponds and reservoirs in rural settings [20–28]. Application has started expanding to *Aedes* habitat detection [29–32]. In Tapachula, Mexico, an urban residential setting, researchers used drones to detect inaccessible *Aedes* larval habitats [26]. In Thailand, researchers used Google Street View images and convolutional neural networks (CNN) to produce density maps of small containers, including buckets and tires [33,34]. However, they cited incomplete coverage, outdated imagery, and inaccessibility of informal settlements by Google cars as limitations.

Here, we build on previous approaches by combining high-resolution drone imagery with supervised machine learning to map potential larval habitats across a dense urban landscape at scale. Using local larval surveillance data to guide our selection of epidemiologically relevant container types, we trained a deep learning model to automatically detect these containers in drone imagery.

## Methods

### Study context

Dar es Salaam is the economic center of Tanzania and the largest city in East Africa [35]. It has a population of 5.38 million people and is situated on the coast of the Indian Ocean by the estuary of the Msimbazi River. Dar es Salaam is rapidly urbanizing with limited institutional capacity to plan and regulate development, with more than 70% living in informal settlements characterized by inadequate service provision [12,36–43]. With unreliable water supply across much of the city, rainwater harvesting and other means of collecting and storing water in various container types are common practices [12,40].

In the last decade, Dar es Salaam has experienced recurrent outbreaks of dengue [16,44–47]. Outside of outbreaks, seroprevalence studies reveal sustained transmission of dengue, chikungunya, and yellow fever in Dar es Salaam, a disease burden likely underestimated due to limited diagnostic capacity [48–53].

### Sample site selection

**Fig 1:**
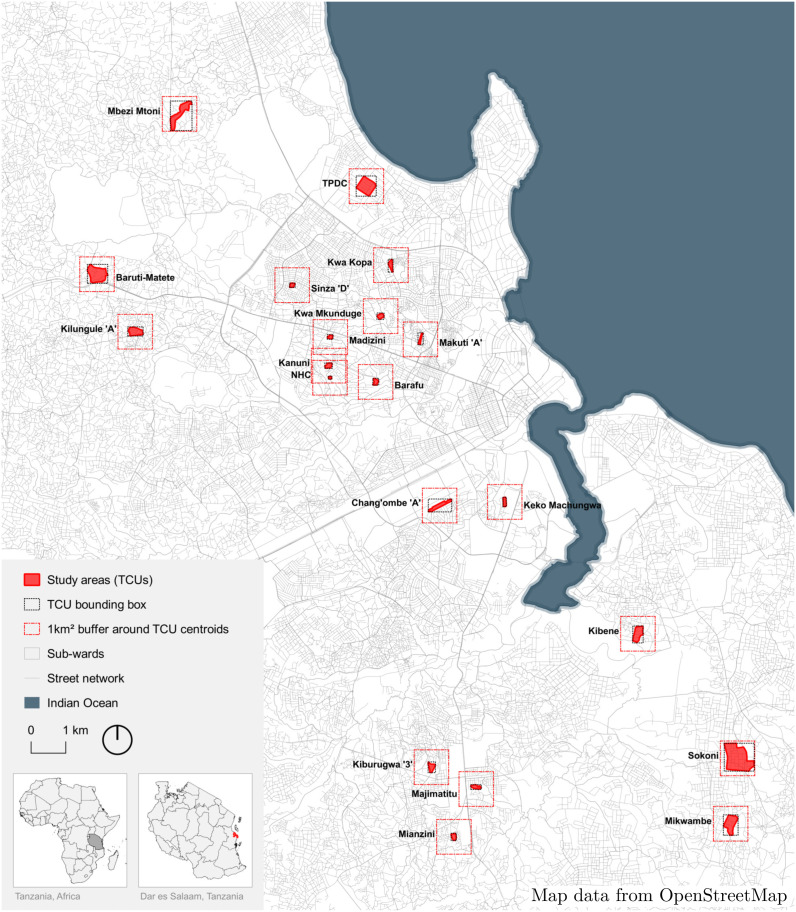
Twenty TCUs selected for inclusion in the study using stratified random sampling. Map of Dar es Salaam illustrating the nested study areas: the study areas (TCUs) in red, the TCU bounding boxes (50m margin around TCU borders) where containers were manually labeled, and a 1 km^2^ square buffer around each TCU centroid where drone imagery was captured. Basemap data from OpenStreetMap [55].

Dar es Salaam has four administrative levels: municipalities, wards, sub-wards, and ten-cell-units (TCUs) [38]. TCUs are the smallest division of the administrative system, originally comprising 10 households and now typically comprising 50–200 households [54]. We used a stratified random sampling approach to select 20 sub-wards for inclusion in our study, ensuring representation of the city’s heterogeneous environment and infrastructure, encompassing informal settlements and average living conditions (Fig 1). To account for this, we excluded sub-wards within 1.5 km of the city center, where there are many high-rise buildings, and peri-urban sub-wards further than 15 km from the city center, where agricultural land use is common. We also excluded sub-wards with drone flight restrictions, including those surrounding the Julius Nyere Airport. Subsequently, we randomly selected one TCU from within each sub-ward for drone and larval surveillance. Our field team approached the local TCU leader of each selected sub-ward to obtain consent to participate in our study. Prior to beginning data collection, we mapped the outer border of each selected TCU using smartphones with GPS capability.

### Larval mosquito surveillance

Over the course of eight months from July 2023 to February 2024, community-owned resource persons (CORPs) conducted larval surveillance in 19 of the 20 drone-surveyed TCUs. CORPs are individual community members appointed and managed through neighborhood health communities, a network established through Dar es Salaam’s Urban Malaria Control Programme [56]. In our study, we hired CORPs to identify all observed natural water bodies and man-made containers, within the defined TCU borders, from a predetermined list of containers of interest. CORPs sampled each potential habitat for mosquito larvae by performing an average of 10 dips with a 350 ml dipper or pipette, according to habitat size. Larva genus was distinguished in the field, reported as *Aedes*, *Anopheles* and/or *Culex*. CORPs sampled all observed habitats, though some may have been missed due to human error. Importantly, only larvae-positive habitats were recorded; habitats without larvae were not. When multiple habitats of the same type were present at a site (for example, several tires), CORPs aggregated larvae and reported them as a single collection. Therefore, each collection represents larval detection at the habitat-type level within a TCU. Larval surveillance served the purpose of confirming container types relevant to *Aedes* larval presence within our study setting.

### Drone imagery collection and labeling

We partnered with Tanzania Flying Labs to systematically capture overlapping drone images across each TCU, which were processed into a single, very-high-resolution orthophoto of each TCU, with permission from each TCU leader. Drone surveys were done in September 2023, within the time frame of larval surveillance, but otherwise unlinked. We used senseFly eBeeX and DJI Phantom 4 Pro drones to capture a 1 km^2^ block, encompassing the TCU and surrounding area. Drones were equipped with senseFlyAeriaX 18.5 (6000 × 4000 pixels), S.O.D.A. 10.6 (5472 × 3648 pixels), and internal DJI Phantom 4 Pro (4864 × 3648 pixels) RGB sensors. We used different types of drones and sensors based on terrain and public safety. We systematically collected overlapping photometric and geo-referenced data and standardized all images during post-processing. Then, we used PIX4D software to generate high-resolution orthophotos, ranging in resolution from 2.68–5.20 cm/pixel, with most image resolutions between 3.10–4.00 cm/pixel.

To standardize shape across all TCUs, we created a bounding box with a 50m margin from the hand-surveyed, irregular TCU border. Using QGIS and following a detailed protocol with example images, we labeled all visible containers of interest, including water tanks, buckets, jerry cans, and tires within the bounding boxes. Across all 20 TCUs, we labeled 9,445 buckets, 3,113 tires, and 2,463 tanks.

### Statistical methods

Because containers without larvae were not recorded, the dataset includes only containers in which at least one mosquito larva genus (*Anopheles*, *Aedes*, or *Culex*) was detected. Consequently, the following analysis should be interpreted as the odds of detecting *Aedes* larvae in a specific container type conditional on the presence of any mosquito larvae. We fit a multi-variable logistic regression model using a generalized linear model with a binomial distribution. The categorical predictor was container type and streams, the most commonly observed larva-positive habitat, were the reference category. We derived odds ratios (OR) and 95% confidence intervals (CI) by exponentiating the model coefficients, and assessed statistical significance using Wald tests. Statistical analyses were conducted in R (Version 2023.12.1 + 402 (2023.12.1 + 402))

Later, to further investigate the machine learning results, we examined the relationship between mapped, predicted container densities and population density. To do so, we calculated Pearson’s correlation coefficient. Population data was obtained from the Humanitarian Data Exchange and the Data for Good at Meta platform. Spatial analyses and maps were conducted in QGIS (version 3.22.4).

### Machine learning methods

We divided labeled drone imagery into training, validation, and test sets with a 75/15/10% ratio, corresponding to 15, 3, and 2 TCUs respectively. The total number of annotated containers in train, val and test sets were 10,487 (6,389 buckets, 2,140 tires and 1,958 tanks), 2,568 (1,806 buckets, 593 tires and 169 tanks) and 1,966 (1,250 buckets, 380 tires and 336 tanks), respectively.

We built a single model for all four selected container classes. To maintain a relatively equal distribution of objects and avoid overlap between training and evaluation sets, we split labels at area-level. The target classes, which are very small items compared to the background, comprised less than 1% of all pixels. Because segmentation models take small input patches as input, all images were cropped into 512 × 512 pixel squares.

We selected a U-Net model, which is a widely-used deep learning model for supervised image segmentation to account for the small size of the target objects [57–59]. The U-Net model enabled pixel-level mapping of small containers in a complex urban environment [60]. We applied the U-Net with an encoder-decoder structure to extract meaningful features and reconstruct images while preserving spatial accuracy. The encoder, based on ResNet-34 [61], reduced image size while capturing essential patterns, and the decoder restored the full-resolution output using skip connections for geometric consistency. This process generated a semantic map and each pixel was assigned an object type (e.g., see [62] for an introduction to deep learning for high-resolution remote sensing imagery). We optimized the model using the AdamW optimization algorithm and a cross-entropy loss function with class frequency weighting to address class imbalance [63–65]. We determined the class weights based on the pixel counts, assigning higher weights to the underrepresented classes, and then adjusted the weights based on the performance on the validation data set. The final class weights used in the loss function were 1, 5, 3, and 1 for the background, buckets, tires, and water tanks, respectively. The model weights were initialized with pre-trained weights from models trained on the Nacala-Roof-Material dataset [66].

We assessed model performance using the validation set during training, and the one with the best macro-averaged Intersection over Union (mIoU) across four classes was selected for final evaluation. Intersection over Union (the IoU is also known as the Jaccard index) measures the overlap between predicted and ground truth regions, ranging from 0 to 1. A higher IoU indicates better alignment, with 1 meaning perfect overlap and 0 meaning no overlap. The macro average mIoU, or the average of the IoUs computed for each class individually, was used to account for class imbalances. Overall accuracy measures the proportion of correctly classified pixels across the entire test set and reflects general performance. However, it can be misleading when class imbalance is present, while mIoU provides a more balanced assessment of segmentation quality. Model hyperparameters were selected based on the best mIoU on the validation set. The selected model was re-evaluated on a separate test set to assess accuracy and IoU at pixel level. The macro average accuracy and individual class-specific accuracy and IoU values were computed. The training process was repeated five times to assess its stability. The test set was not used in any step of the model creation process. Predicting all potential larval habitats in the study area, which spans approximately 27.27 km^2^, took 81 minutes. An AMD MI250X GPU with 64GB of VRAM provided by LUMI (https://lumi-supercomputer.eu/) was used for predictions. Approximately 1,000 GPU hours were spent on hyperparameter tuning and training.

## Results

**Fig 2:**
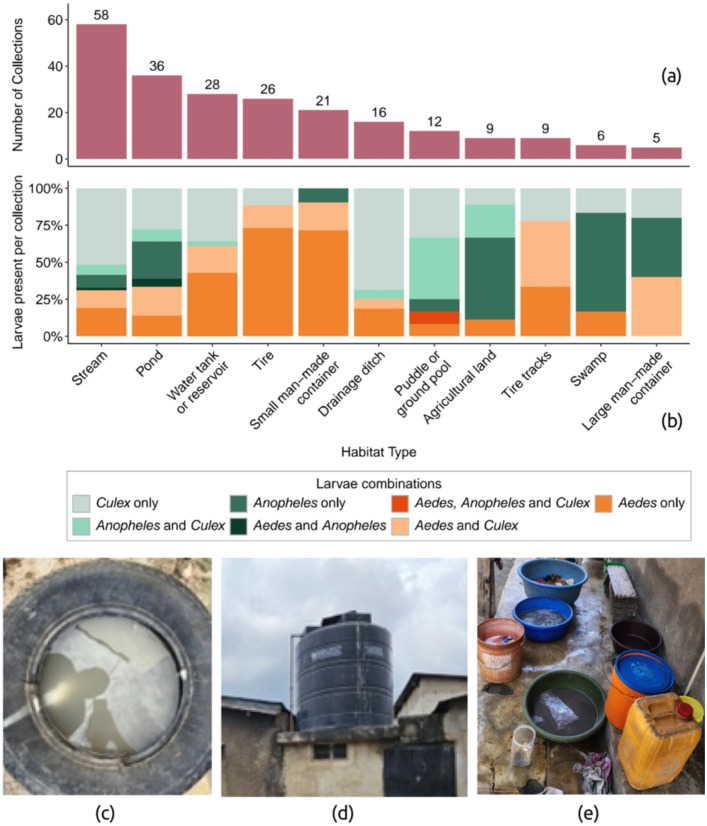
Larval surveillance across 19 TCUs of Dar es Salaam. **a)** Number of collections from each habitat type across Dar es Salaam from July 2023 to February 2024. We defined a collection as a single sampling event. An event could include larvae from a single habitat or pooled larvae from multiple habitats of the same type within a single site (i.e., one bucket or all buckets observed at a household). **b)** Proportion of larval genus found in each habitat type across all collections. Photos show c) a water-filled tire, d) a rooftop water tank, and e) water-filled buckets (small man-made containers).

*Aedes* larvae were detected in 10 different habitat types, including natural and man-made, during eight months of larval surveillance across 19 TCUs of Dar es Salaam (Fig 2a,b). Among habitats with any mosquito larvae present, the odds of detecting *Aedes* larvae was significantly higher in small man-made containers (OR=19.5, 95% CI:[4.98−131.0], *p* < 0.001), tires (OR=15.7, 95% CI:[4.75−72.4], *p* < 0.001), tire tracks (OR=7.18, 95% CI:[1.56−51.44], *p* = 0.02), and water tanks or reservoirs (OR=3.17, 95% CI:[1.26−8.29], *p* = 0.02). The primary types of small man-made containers surveyed were buckets and jerry cans. Additionally, *Aedes* larvae were occasionally found in drainage ditches and natural water bodies (e.g., ponds, puddles, agricultural land, swamps).

**Fig 3:**
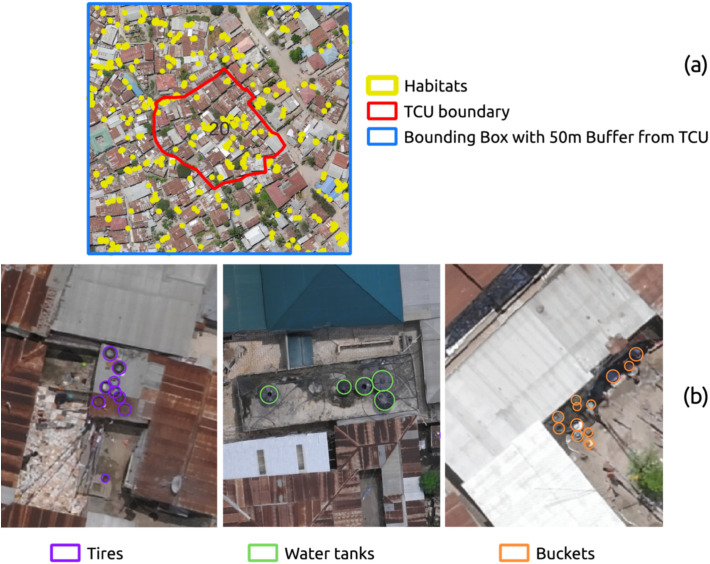
Manually annotated drone imagery to identify potential larval container habitats. **a)** Aerial perspective at 3–5 cm/pixel resolution showing widespread distribution of potential *Aedes* larval habitats, including tires, buckets, jerrycans, and water tanks. **b)** Discarded tires and water tanks are observed on rooftops, and clusters of buckets are observed in peri-domestic spaces.

To explore the spatial distribution of key potential larval habitats with increased odds of harboring *Aedes* larvae, we used drone imagery to map tires, small man-made containers (buckets and jerrycans), and water tanks (Fig 2c-e). Although identified as having increased odds of *Aedes* larval presence, tire tracks were inconclusively visible in our drone imagery and thus excluded from the study. The aerial perspective revealed potentially overlooked habitats, including discarded tires on rooftops, unsealed water tanks (no top lid), and buckets in enclosed peri-domestic spaces (Fig 2d). Across a cumulative area of 4.60 km^2^ within 20 bounding boxes, each encompassing an individual TCU, we labeled a total of 9,445 buckets or jerrycans, 3,113 tires, and 2,463 water tanks (see Fig 3).

### Mapping potential container habitats at scale using a machine learning model

To estimate habitat presence on a larger scale, we developed a U-Net semantic segmentation model with ResNet-34 encoder to predict these three key potential larval habitats in unlabeled drone imagery. Total drone coverage across both labeled and unlabeled areas, encompassing all 20 TCUs surveyed, was 27.27 km^2^. Within the available drone imagery and outside of the bounding boxes, an area equal to 22.67km^2^, we predicted a total of 135,950 containers (buckets, tires, and water tanks). The U-Net model segmented connected objects as a single segment. To avoid under-prediction, the predicted area was converted to predicted counts by dividing the total segmented area of each class by the average area of labeled objects of this class.

**Fig 4:**
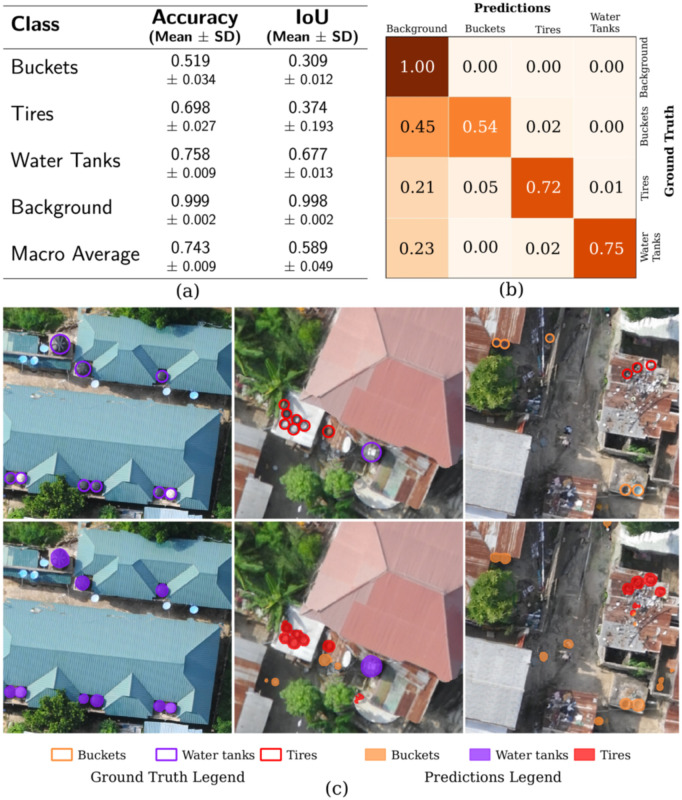
Model predictions. **(a)** The table presents the mean accuracy and IoU scores across five trials, along with their standard deviations. **(b)** The confusion matrix shows the proportion of correctly classified pixels for each class (along the diagonal) and those mistakenly classified. Darker shades of red indicate higher accuracy. The values are based on a single trial and are rounded to two decimal places. **(c)** Manually labeled habitats (top row) and model predictions in the validation set (bottom row).

The U-Net model detects small containers with an overall accuracy score of 0.743 and an mIoU of 0.589 based on the test set. The mean (± standard deviation) accuracy of buckets, tires, and water tanks over five trials is 0.519 ± 0.034, 0.698 ± 0.027, and 0.758 ± 0.009, respectively (see Fig 4). We trained the same model five times to evaluate whether the performance is stable and not significantly affected by the randomness of the training process (due to random image augmentations, mini-batch selection, and weight initialization). The low standard deviation across five trials suggests that it is stable (see Fig 4).

**Fig 5:**
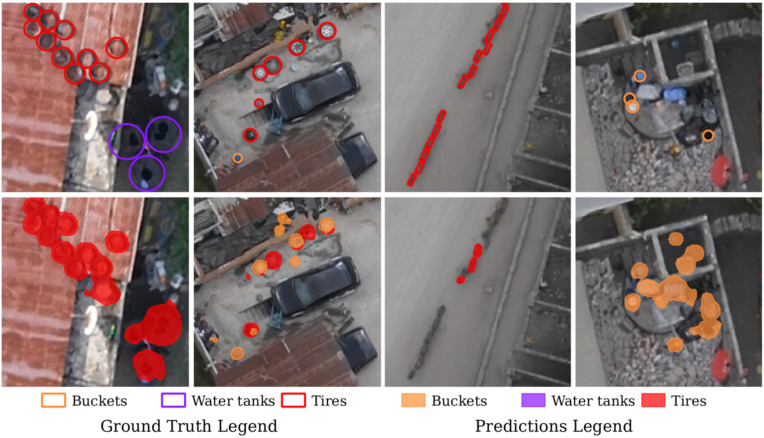
Errors in predictions. Top row shows manual annotations of water tanks, tires and buckets or jerrycans and the bottom row shows predictions from the U-Net model on the test set. In the first column, water tanks are detected as tires and in the second column, tires with rims are detected as buckets. The third column shows tires used for fencing missed, and the fourth column shows sinks and other objects incorrectly detected as buckets.

Our model tended to over-predict buckets and jerrycans, likely due to both these object’s variability in form and similarity to other objects. False detections included small circular items like sinks and hubcaps (see Fig 5). On the contrary, water tanks and tires are fairly uniform in size and form, and the model predicted these with more accuracy. Discarded tires used as fencing were sometimes missed because of their rectangular form when viewed from an aerial perspective, compared to the majority of ring-shaped tires laying on their sides. As it pertains to our study, these tires are less of a concern because they are often partially buried and filled with sand, making them irrelevant as potential larval habitats. Sometimes, the model misidentified large, dark puddles as water tanks.

**Fig 6:**
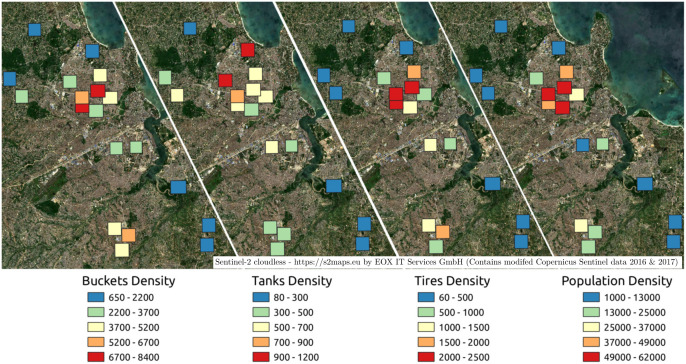
Density map of predicted containers across 20 TCUs of Dar es Salaam. Containers and population densities are mapped as objects or people/km^2^ and categorized into 5 ranges, where red represents highest density and blue lowest. Regions highlighted in red indicate areas of higher container density. Tire and bucket densities are highest closest to the city center, whereas water tank density is greater in TCUs northwest of central Dar es Salaam. *[Basemap from Sentinel-2 cloudless -* https://s2maps.eu *by EOX IT Services GmbH (Contains modified Copernicus Sentinel data 2016 & 2017)]*.

We hypothesized that container density would positively correlate with population density based on the assumption that more people leads to accumulation of more things. To test this, we mapped the predicted density of each priority container type at the TCU level (covering the entire drone image) and examined it in relation to aggregated population density (see Fig 6).

By Pearson’s correlation test, the densities of tires and buckets are significantly associated with population density. As population density increases, the density of tires and buckets increases. However, the density of water tanks did not correlate with population density, which suggests another factor, possibly socioeconomic status, drives their distribution. TCUs farther from the city center, with lower population density, had lower densities of predicted containers (see Fig 6). Central TCUs including Madizini, Kwa Mkunduge and Kanuni had the highest densities of predicted tires. Kwa Mkunduge and Kanuni had the highest densities of predicted buckets/jerrycans. TPDC and Sinza ‘D’ had the highest densities of predicted water tanks, both TCUs locally known to have higher socioeconomic status [67].

## Discussion

This study demonstrates the feasibility of drone imagery and supervised machine learning as a remote, scalable tool to map potential *Aedes* larval container habitats at very high resolution. The resulting maps could guide ground-based larval surveillance and larval source management by highlighting areas with a high density of containers for further investigation. Three key habitat types: water tanks, tires and buckets/jerrycans, can be detected at scale with up to 75% accuracy using a U-Net model. The aerial perspective of drone imagery revealed the abundance of potential container habitats distributed heterogeneously across TCUs, including possibly overlooked cryptic habitats like discarded tires on rooftops.

These rooftop tires can easily collect rainwater and are likely undisturbed, creating favorable conditions for *Aedes* larvae to proliferate [68]. In South Asia and South America, rooftop tires have been associated with a disproportionately high percentage of total positive containers compared with other larval habitats [68,69]. In south-eastern Tanzania, discarded tires were found to be the most commonly infested habitat [70]. We also observed that many water tanks were stored on rooftops. In Pakistan and Brazil, rooftop water tanks have been identified as higher-risk for *Aedes* larvae presence [69,71].

### Machine learning performance

The machine learning model demonstrated the ability to detect small objects at scale from high-resolution drone imagery. Detection accuracy was higher for tires and water tanks than for buckets and jerrycans, likely due to the relatively standardized size and form of these objects, making them easier for the model to recognize. In contrast, buckets and jerrycans vary widely in shape and size and may resemble other small objects such as discarded materials, puddles, outdoor sinks and household items, which reduces machine learning discriminability. As commonly observed in many semantic segmentation studies, the training dataset exhibited strong class imbalance, with pixels representing the background far outnumbering those corresponding to containers (99.84% background, 0.02% buckets, 0.03% tires, and 0.11% water tanks). This imbalance initially posed challenges during training, but performance improved after applying stronger weights in the cross-entropy loss function. Detection accuracy for buckets and jerrycans may be further improved through expanded labeled datasets, more granular annotation strategies, increased spatial resolution by capturing drone imagery at a lower altitude with the same AeriaX sensor, and improved orthorectification techniques. While buckets in our study had higher odds of *Aedes* larval presence compared with streams with any type of larvae present, previous studies have shown that although buckets are common, only a small proportion of the total number of buckets are typically infested with *Aedes* larvae [68]. Overall, buckets may be less epidemiologically relevant than other container types.

### Remaining challenges

A limitation of our study is the absence of ground-truth container counts to validate the manually labeled containers identified in drone imagery. As manual annotation was used to generate the training labels for the machine learning model, inaccuracies in these labels could introduce bias in model training and predictions. Given the very high resolution of the drone imagery, manual labels are likely accurate; however, conducting ground-based surveys in selected areas to count and verify containers would strengthen this assumption. Secondly, we did not conduct entomological sampling to determine whether mapped containers were actually positive for *Aedes* larvae. As a result, the presented maps of container densities should not be interpreted as risk maps of *Aedes* larvae; this study does not establish a relationship between labeled containers and the presence of mosquito larvae. Previously, a household survey in Kinondoni district in Dar es Salaam found that more than 60% of inspected water-holding containers contained *Aedes* larvae or pupae, highlighting the importance of domestic containers in sustaining dengue vector populations [15]. Future entomological sampling to determine container indices for *Aedes* larvae across buckets, tires, and water tanks, where both positive and negative containers are recorded, would inform prioritization of container management. Longitudinal surveillance would further deepen understanding of *Aedes* larval habitats [72]. However, these data would not be sufficient to translate to container-level infestation maps at the scale presented in this study.

Our drone images captured one snapshot in time; capturing repeated drone images over time would provide insight into the temporal dynamics of potential container habitats. Of note, we focused on containers identified to be of relevance in an urban sub-Saharan African setting and acknowledge this may vary in Asia and the Americas. However, the principles of this approach can be adapted to other settings, using local drone imagery annotated to identify the context-specific containers of interest.

High-resolution drone imagery raises privacy concerns. To address this, Tanzania Flying Labs, a local organization, was engaged to lead the drone survey work, promoting community involvement and increasing local acceptability. Drones for mosquito surveillance have received community acceptance in other settings, and both community engagement and ethics are key to consider when implementing any kind of high-resolution drone-based surveillance [73].

### Future investigations and public health implications

This approach could complement ground-based surveillance by providing field teams with maps to understand the spatial distribution and density of high-priority containers. Locating all potential habitats through traditional ground surveillance is labor-intensive and logistically challenging [13,74,75]. In the context of an outbreak, our approach could potentially be used to identify areas with high container densities, guiding ground surveillance work. However, the applicability in this context rests on the assumption that disease transmission in one area is linked to *Aedes* larval presence in that same area. Adjacent research has shown that proximity to *Anopheles* larval habitats is a known risk factor for urban malaria [76].

Environmental management through larval source reduction requires buy-in from the whole community, to establish habits of covering, regular cleaning and emptying of water storage containers that could become *Aedes* larval habitats [77]. Community-based programs have been successfully implemented as a strategy in controlling *Anopheles* mosquito habitats [78–82]. If sustained funding is available, CORPs could implement larval source reduction across Dar es Salaam in TCUs with high densities of containers to reduce potential habitats and engage communities on behavioral interventions like regular container emptying and cleaning [56]. Meta-analysis shows that community-based larval source reduction alone has an inconclusive to positive effect on reducing arboviral disease prevalence, and when combined with larvicidal treatment, a positive effect [83].

To further understand how larval source reduction could be implemented, future studies could investigate whether most identified containers are in public or domestic spaces. This distinction would have implications for intervention design; clean-up of containers in public spaces could be government-led while domestic spaces would need to be community-led. Rooftop containers, which are not easily accessible, present an added challenge as they are unlikely to be reached by routine household-level source reduction efforts. Drone-based larvicide application has been investigated for *Aedes* control, a method which could help access difficult-to-reach habitats [84]. For discarded tires on rooftops, physical interventions such as puncturing to allow water drainage or covering with fine mesh may offer more practical, low-barrier solutions if acceptable.

We examined possible drivers of the heterogeneous spatial distribution of key potential container habitats at the neighborhood level. We hypothesize that water accessibility and socioeconomic status influence the presence and distribution of buckets, tires, and water tanks. In areas with unreliable piped water supply, households commonly store water for domestic use, which unintentionally creates potential larval habitats for *Aedes* mosquitoes [15]. Given that container types showed distinct spatial patterns consistent with different underlying drivers, targeted rather than blanket larval source reduction strategies may be more operationally effective.

## Conclusion

We demonstrate a scalable, high-resolution drone-based surveillance approach for mapping containers that are potential *Aedes* larval habitats across dense urban environments. Resulting maps of predicted container densities across Dar es Salaam highlight the spatial heterogeneity and widespread distribution of these items, potentially driven by population density or underlying risk factors. Further, high-resolution drone imagery revealed discarded tires on rooftops, which may be overlooked during ground-based field surveillance. This approach fills an important surveillance gap in urban environments characterized by informal settlements with limited accessibility. Public health agencies could integrate the density maps into routine surveillance workflows to prioritize field inspections, optimize larval source reduction campaigns, and allocate resources more efficiently.
